# Sugars take a central position in plant growth, development and, stress responses. A focus on apical dominance

**DOI:** 10.3389/fpls.2014.00313

**Published:** 2014-06-30

**Authors:** Wim Van den Ende

**Affiliations:** Laboratory of Molecular Plant Biology, Biology, Katholieke Universiteit LeuvenLeuven, Belgium

**Keywords:** sugar signaling, apical dominance, auxin response, trehalose 6-phosphate, sucrose, phloem transport, hormone signaling

In plant glycobiology, free “metabolic” carbohydrates consist of small sugars (glucose, fructose, sucrose, trehalose), sucrose-derived oligosaccharides (fructans, Raffinose Family Oligosaccharides), starch and its breakdown products (Van den Ende, 2013). All these carbohydrates are directly or indirectly derived from photosynthesis. Plants typically accumulate higher carbohydrate levels as compared with other multicellular eukaryotes, especially when (milder) stresses compromise growth more than photosynthesis (Van den Ende and El-Esawe, in press).

In a historical perspective, the central role of sugars was already suggested many decades ago, with proposed roles in overall plant growth and development (Allsopp, 1954), disease susceptibility (Horsfall and Dimond, 1957), flowering (Kraus and Kraybill, 1918) and apical dominance (Loeb, 1924). The latter two processes were suggested to be regulated by the “nutrient diversion hypothesis” (Gregory and Veale, 1957; Corbesier et al., 1998). This theory, narrowed down to sugar nutrients, states that a minimal level of sugar assimilates needs to reach the apex (flowering) or lateral bud (removal of apical dominance) before floral transition or lateral bud outgrowth is initiated. The discovery that indole-3-acetic acid (IAA) repressed lateral bud outgrowth in decapitated shoots (Thimann, 1937) boosted plant hormone research at the expense of sugar-centered research. However, things changed when small sugars, similar to hormones, were considered as important signals in plants (Moore et al., 2003). Since then, a renewed and strongly increasing worldwide interest in sugar signaling, sensing and metabolism was noticed (Ruan, 2014; Smeekens and Hellmann, 2014).

Mason et al. (2014) focused on the underlying mechanisms involved during apical dominance in pea. The authors challenge a long-held dogma in plant physiology, proposing that sugar signals, and not IAA, initiates lateral bud outgrowth after apex decapitation. For decades, textbooks declare that phloem-mediated IAA transport fluxes down the stem decrease after decapitation, relieving the IAA-mediated inhibition on bud outgrowth. However, the discovery that stem IAA cannot enter the bud, and the fact that IAA application on decapitated stems cannot always prevent bud burst, suggested that a positive signal could overrule IAA-mediated inhibitory effects (Mason et al., 2014).

Buds from the decapitated apex could be released starting from 2.5 h post-decapitation as shown by time-lapse photography (Mason et al., 2014). Importantly, this was not accompanied by a measurable IAA depletion in the adjacent stem. However, a much slower bud release was reported before (Wardlaw and Mortimer, 1970). Mason et al. (2014) designed an elegant set of physiological experiments demonstrating that leaves are the source of a rapid decapitation induced signal that promotes bud release. They reasoned that sucrose could be a candidate for this signaling role. Subsequently, [^11^C] CO_2_ was fed to leaves and the movement of [^11^C] photo-assimilates was monitored along the stem. They found a speed of 150 cm h^−1^, which agreed with the timing of bud burst and phloem-mediated transport. Exogenous sucrose applications promoted bud burst. Moreover, in plants decapitated low on the stem, leaf removal caused a serious delay in bud release, and this could be rescued by feeding sucrose, but not sorbitol, via the petiole. Unfortunately, the sucrose-specific character of this response was not tested. Comparing glucose and sucrose responses could be informative to discriminate between glucose- or sucrose-mediated signaling events.

These data strongly suggest that a minimal threshold sucrose level is required to sustain lateral bud outgrowth. Initiation of bud outgrowth would not make much sense if not enough C would be available to sustain the later stages of bud growth. However, this leaves us with a remaining question: is there still room for hormone signaling events in this process?

I speculate that the answer on this question is “yes.” Internal sugar/IAA ratios within lateral buds or within the adjacent stem may be somehow integrated prior to lateral bud outgrowth initiation. In my opinion further studies should answer the following crucial questions (i) Are increased sugar levels associated with changed hormone levels in buds and how does this change over time? (ii) Do bud IAA levels depend on IAA biosynthesis from tryptophan within the bud, or is indol-3-aldehyde, a phloem-mobile lateral bud inhibitor (Nakajima et al., 2002), involved via a mechanism that perhaps depends on sugar signaling? If so, can the presence of indol-3-aldehyde in the phloem sap be confirmed? (iii) Alternatively, could phloem residing Aux/IAA transcripts, involved in the regulation of auxin signaling, enter the bud followed by differential translation depending on the actual sugar status? Such transcripts may be important, since their upregulation in the phloem influences both root and shoot branching, as well as overall IAA sensitivity (Golan et al., 2013). Some of these views fit with emerging evidence that sugars can control auxin levels in plants (LeClere et al., 2010; Sairanen et al., 2012). Hexokinase (HXK) mediated sugar signaling may be central in such processes, since AtHXK1 overexpressors relieved their apical dominance (Kelly et al., 2012). This was associated with lowered expression levels of genes that encode crucial players in auxin signaling, suggesting that glucose signals control downstream auxin signaling in Arabidopsis (Kelly et al., 2012).

It cannot be excluded that light dependent signaling mechanisms, or any other positive factors, may have remained undetected in the pea apical dominance paper (Mason et al., 2014). Moreover, these experiments should be repeated in an array of other plant species, before any general conclusions can be derived that would apply to all higher plants. GA, sugars and light play crucial roles during bud outgrowth in roses through increasing sugar demand, by upregulating the expression and activity of vacuolar invertases (Choubane et al., 2012; Rabot et al., 2012).

Despite these critical notes, it should be recognized that the paper of Mason et al. (2014) boosts further research in both hormone and sugar signaling communities, by stimulating hormone workers to consider sugars and vice-versa. So far, attempts to integrate sugar and hormone signaling events are rather scarce (Bolouri Moghaddam and Van den Ende, 2013). The putative importance of sugar signaling events in apical dominance ads to a list of other physiological processes that are believed to be controlled by sugars or their phosphorylated derivatives such as trehalose 6-phosphate (T6P), which has been suggested as an important indicator of the carbohydrate status in plants and negative regulator of SnRK1 (Zhang et al., 2009). The latter is a central player in overall energy homeostasis (Baena-González et al., 2007) together with TOR kinase (Robaglia et al., 2012; Xiong et al., 2013). The T6P/SnRK1 module is involved in sugar signaling processes (Baena-González et al., 2007) together with sucrose-specific DELLA-mediated processes (Li et al., 2014) controlling, for example, anthocyanin accumulation as part of the plants defense response (Nakabayashi et al., 2014).

In line with the earlier ideas of Allsopp (1954), recent molecular and biochemical evidences revealed that sugars regulate the juvenile-to-adult phase transition by modulating miR156 expression, with possible involvement of HXK-mediated signaling (Duarte et al., 2013; Yang et al., 2013; Yu et al., 2013). Moreover, it was demonstrated that rhythmic, endogenous sugar signals, independently of light signals, entrain circadian rhythms by regulating the expression of circadian clock components (Haydon et al., 2013). Recent data strongly suggest that leaf diurnal starch dynamics (Graf and Smith, 2011) intimately connect with T6P levels (Martins et al., 2013). In line with the nutrient diversion hypothesis, it is proposed that plants sense the T6P status prior to the transition to flowering (Wahl et al., 2013). Photoperiod modification of starch homeostasis by CONSTANS, a stimulator of the FLOWERING LOCUS T, may be crucial for increasing the sugar mobilization demanded by the floral transition (Ortiz-Marchena et al., 2014). These observations urge further research into T6P levels, starch dynamics and SnRK1 activities in the context of apical dominance.

Clearly, we are only at the beginning of our understanding of the complexity of cellular sugar homeostasis, and deciphering how this exactly connects to hormonal regulatory mechanisms is an important challenge.

## Conflict of interest statement

The author declares that the research was conducted in the absence of any commercial or financial relationships that could be construed as a potential conflict of interest.
